# Supplementary material for: Modelling Skylarks (Alauda arvensis) to Predict Impacts of Changes in Land Management and Policy: Development and Testing of an Agent-Based Model
Source: PLoS One. 2013 Jun 6;8(6):e65803. doi: 10.1371/journal.pone.0065803 (PMC3675089; doi:10.1371/journal.pone.0065803)
Supplement: Supporting Information S4 — The skylark ODdox as a zipped archive. (ZIP) [file pone.0065803.s004.zip › Skylark_ODdox/functions_func_0x72.html]

ALMaSS Skylark ODdox: Class Members - Functions


ALMaSS Skylark ODdox
 2.0


- Main Page
- Related Pages
- Classes
- Files

- Class List
- Class Index
- Class Hierarchy
- Class Members

- All
- Functions
- Variables
- Typedefs

- a
- b
- c
- d
- e
- f
- g
- h
- i
- j
- k
- l
- m
- n
- o
- p
- r
- s
- t
- u
- v
- w
- y
- z
- ~

### - r -

- Railway()
  : Railway
- Raining()
  : Weather
- RandomLocation()
  : Landscape
- RandomVegStartValues()
  : VegElement
- RasterMap()
  : RasterMap
- ReadBugPercentageFile()
  : VegElement
  , CropData
- ReadInput()
  : Landscape
- ReadPolys()
  : Landscape
- ReadRotation()
  : Farm
- ReadSymbols()
  : Configurator
  , Landscape
- RecalculateBugsNStuff()
  : LE
  , VegElement
- RecordRodenticidePolyData()
  : RodenticideManager
- ReduceMass()
  : BaitLocation
- ReduceVeg()
  : LE
  , VegElement
- ReduceVeg\_Extended()
  : LE
  , VegElement
- ReduceWeedBiomass()
  : LE
  , VegElement
- ReEvaluateTerritory()
  : Skylark\_Male
- Register()
  : Configurator
- ReHouse()
  : Skylark\_Population\_Manager
- RemoveEM()
  : Skylark\_Adult
- RemoveFemale()
  : SkTerritories
- RemoveField()
  : FarmManager
  , Farm
- RemoveMale()
  : SkTerritories
- Reset()
  : BaitLocation
  , Calendar
- ResetBreedingSuccess()
  : Skylark\_Female
- ResetTrace()
  : LE
- River()
  : River
- RiversidePlants()
  : RiversidePlants
- RiversideTrees()
  : RiversideTrees
- RoadsideVerge()
  : RoadsideVerge
- RodenticideManager()
  : RodenticideManager
- RowCultivation()
  : Farm
- Run()
  : Population\_Manager
- RuralResidential()
  : RuralResidential


- Generated on Thu Jan 10 2013 13:15:39 for ALMaSS Skylark ODdox by
   1.8.1.1
